# Formulation and Evaluation of Isorhamnetin SNEDDS for Neuroprotection in Alzheimer's Disease

**DOI:** 10.1002/alz70859_102526

**Published:** 2025-12-25

**Authors:** Sambita Panda, Sachin Kumar Singh, Bushra Bashir, Biswajit Pal, Sukriti Vishwas, Jaskiran Kaur

**Affiliations:** ^1^ Lovely Professional University, Phagwara, Punjab India; ^2^ School of Pharmaceutical Sciences, Lovely Professional University, Jalandhar, Punjab India; ^3^ Lovely Professional University, Jalandhar, Punjab India

## Abstract

**Background:**

Alzheimer's disease (AD) is a progressive neurodegenerative disorder characterized by memory loss, cognitive decline, and behavioral changes. The disease is marked by the accumulation of amyloid‐beta plaques and tau tangles, which disrupt neuronal function and lead to cell death. Neuroinflammation, oxidative stress, and synaptic dysfunction further contribute to AD pathology. Various studies evaluate the therapeutic properties of the phytoconstituents like curcumin, piperine, resveratrol, berberine and quercetin for the treatment of AD. Among these phytoconstituents, Isorhamnetin (IH) is reported to have neuroprotective effects against AD by inhibiting the expression of p‐JNK, p‐p38, and p‐NFκB proteins. Studies showed that IH mediates a protective effect against oxidative stress and neuroinflammation. Despite its prospects, the therapeutic use of IH at target sites is limited due to their poor solubility, less bioavailability and poor blood brain barrier (BBB) penetration.

**Method:**

To address these limitations, a IH loaded self‐emulsifying drug delivery system (SNEDDS) was developed which improved drug loading, stability, bioavailability, and BBB permeability. Further, pharmacodynamic study were conducted using Morris Water Maize test to assess the cognitive performance of rats. Additionally biochemical assessment were performed to measure key markers of AD.

**Result:**

The optimized IH‐loaded SNEDDS formulation demonstrated a droplet size of 59.46 nm, polydispersity index of 0.3, zeta potential of ‐19 mV, and 96% drug loading respectively. Pharmacodynamic evaluations showed significant improvements in cognitive and motor functions in rats at high doses. Biochemical analysis revealed that the formulation effectively reduced AChE levels, amyloid‐beta, oxidative stress, and neuroinflammation.

**Conclusion:**

IH‐loaded SNEDDS served as a therapeutic approach in the management of AD

